# Atropine Differentially Modulates ECM Production by Ocular Fibroblasts, and Its Ocular Surface Toxicity Is Blunted by Colostrum

**DOI:** 10.3390/biomedicines8040078

**Published:** 2020-04-05

**Authors:** Martina Cristaldi, Melania Olivieri, Salvatore Pezzino, Giorgia Spampinato, Gabriella Lupo, Carmelina Daniela Anfuso, Dario Rusciano

**Affiliations:** 1 Research Center, Sooft Italia SpA c/o Biologic Tower, University of Catania, 95123 Catania, Italy; martina.cristaldi@sooft.it (M.C.); melania.olivieri@sooft.it (M.O.); salvatore.pezzino@sooft.it (S.P.); giorgia.spampinato@sooft.it (G.S.); 2 Department of Biomedical and Biotechnological Sciences, Biologic Tower, University of Catania, 95123 Catania, Italy; gabriella.lupo@unict.it

**Keywords:** Atropine, 7-methyl-xanthine, myopia, collagen, sclera, choroid, colostrum, 2-fucosyl-lactose, fibroblasts

## Abstract

Background: The etiology and the mechanism behind atropine treatment of progressive myopia are still poorly understood. Our study addressed the role of scleral and choroidal fibroblasts in myopia development and atropine function. Methods: Fibroblasts treated in vitro with atropine or 7-methylxanthine were tested for ECM production by Western blotting. Corneal epithelial cells were treated with atropine in the presence or absence of colostrum or fucosyl-lactose, and cell survival was evaluated by the MTT metabolic test. Results: Atropine and 7-methyl-xanthine stimulated collagen I and fibronectin production in scleral fibroblasts, while they inhibited their production in choroidal fibroblasts. Four days of treatment with atropine of corneal epithelial cells significantly decreased cell viability, which could be prevented by the presence of colostrum or fucosyl-lactose. Conclusions: Our results show that atropine may function in different ways in different eye districts, strengthening the scleral ECM and increasing permeability in the choroid. The finding that colostrum or fucosyl-lactose attenuate the corneal epithelial toxicity after long-term atropine treatment suggests the possibility that both compounds can efficiently blunt its toxicity in children subjected to chronic atropine treatment.

## 1. Introduction

Myopia is an aberrometric defect of vision well defined in its anatomy and physiology, although its etiology is not completely clarified. High myopia, which develops starting from a very young age, is a serious risk factor for further eye pathologies such as glaucoma, choroidal ischemia, and retinal detachment.

Myopia most often results from the abnormal elongation of the eye globe, bringing the retina out of focus [1]. Several clinical trials have shown a clear effect of atropine in controlling myopia progression in children at risk of developing an invalidating disease [2]. Chronic atropine treatment, as it is required for the control of progressing myopia, may have side effects related to a persistent mydriatic activity, such as photophobia and poor near visual acuity, or unrelated, such as allergy. In each case, it has been found that low-dose atropine (<0.05%) generates much lower side effects and still gives a good control response on myopia progression [3]. For this reason, low-dose atropine is currently used as a galenic, off-label treatment in this category of subjects. However, the molecular mechanisms behind high myopia development and the reason atropine works in slowing down its progression are not fully understood. Atropine, extracted from *Atropa belladonna*, is a non-selective muscarinic acetylcholine receptor (mAChR) antagonist. The human sclera is composed of 90% heterologous collagen fibrils mainly represented by collagen type 1, and its resident cells express all five muscarinic receptors (M1–M5) [4]. There are at least two possible hypotheses to explain the abnormal elongation of the eye globe. The first hypothesis (which is the most credited), which we may call passive elongation, evokes a reduced thickness and/or rigidity of the extracellular matrix (ECM) of the sclera, leading the eye globe to prolapse posteriorly [5,6], similarly to what happens in keratoconic corneas [7]. In this case, atropine would be expected to induce a reinforcement of the sclera, by increasing its ECM elements biosynthesis. The second hypothesis (opposite to the first), which we may call active elongation, suggests that an increased synthesis of ECM constituents (because of an overstimulation possibly due to genetic and/or environmental factors), may finally result in an elongated, myopic eye. In this case, an efficient preventive treatment should reduce ECM components biosynthesis.

In fact, different authors describe in myopic eyes a thinning of the sclera, which becomes more extensible and thus favors eyeball elongation. Such remodeling of the sclera in myopia is associated with decreased amounts of collagen type 1 (the main component of its ECM), and treatment with atropine in several in vivo studies has been shown to increase the content of collagen-1 in sclera ECM. Another relevant correlate with myopia development is a thinning of the choroid and a decrease of blood perfusion of eye tissues [8,9].

We present here data showing that atropine may indeed work in two different ways. When used on a human scleral fibroblast cell line (HSF), it appears to increase collagen type I and fibronectin biosynthesis, in agreement with the “prolapse” hypothesis. When used on a human choroid fibroblast cell line (HOCF) it appears to decrease collagen type I and fibronectin biosynthesis. Coherently with these findings, we show that 7-methylxanthine—also reported to be able to counteract myopia progression [10,11]—also stimulates collagen and fibronectin production in HSF cells, while it decreases ECM synthesis in HOCF cells.

Finally, the long-term use of atropine eye drops in children might increase the risk of dry eye [12]. Indeed, we show that prolonged treatment in vitro of human epithelial corneal cells (HCE-F) with low dose atropine dramatically decreases cell viability. However, such toxicity could be efficiently blunted by either colostrum or by its main oligosaccharide, 2-fucosyl-lactose (2FL).

## 2. Materials and Methods

### 2.1. Cell Culture

Human Scleral Fibroblasts (HSF) were purchased from Lifeline Cell Technology (Frederick, Maryland, USA) and cultivated in Lifeline FibroLife medium supplemented with FibroLife S2 LifeFactors Kit (ibid., Cat. no. LL-0011). Human choroidal fibroblasts (HOCF) were purchased from Science-cell (Carlsbad, California, USA) and cultivated in Fibroblast Medium kit (ibid., Cat. No. 2301). HSF and HOCF were grown at 37 °C, in a humidified atmosphere containing 5% CO_2_. Human corneal epithelial cells (HCE-F) were derived by dispase II and trypsin treatment from the epithelial layer of a human corneal button of a donor patient after keratoplastic surgery (Cornea, in press). They were grown in DMEM-F12 (Gibco – Thermo Fisher Scientific, Massachusetts, USA, Cat. No. 12634010) containing 2% FBS (Sigma Aldrich, St. Louis, Missouri, USA, Cat. No. F7524), supplemented with factors from the corneal epithelial cell growth kit (ATCC, Manassas, Virginia, USA, Cat. No. PCS-700-040).

### 2.2. ECM Proteins Analysis by Western Blotting

HSF or HOCF cells were seeded at a density of 5 × 10^5^ cells/well in a six-well plate, and left to adhere overnight in the incubator. Cells were starved 4 h in SFM and then the following treatments were carried out in SFM: atropine (Cat. No. 40489627, MOLEKULA, Italy) 100 μM, 250 μM, 500 μM, and 1 mM, or 7-methyl-xanthine (Cat. No. 18393522, MOLEKULA, Italy) 100 μM, 250 μM, and 500 μM, for 24 and 48 h. The evaluation of dose-effect with atropine arrives at 1 mM (0.03%), because this is an average therapeutic dose given to patients to try and stop their progressing myopia [2], and the dose tested for toxicity on corneal epithelial cells (see below). Treatments with 7MX arrived at 0.5 mM because at this dose we already see a maximal effect, and 7MX is given as an oral supplement, thus there is no clear dose for in vitro studies. At the end of the incubation period with either atropine or 7MX, cells were lysed in RIPA extraction buffer (Calbiochem-Merck, Darmstadt, Germany, Cat. No. 20188) with a protease inhibitors cocktail (Protease Inhibitor Cocktail Set III EDTA-Free, Calbiochem-Merck, Darmstadt, Germany, Cat. No. 539134) as described previously [13]. Briefly, cell monolayers were rinsed 3 times with cold PBS, and extracted for 30 min on ice. The extract was centrifuged at 13,000 rpm for 20 min at 4 °C, and proteins in the supernatant quantitated by the BCA protein assay. For Western blot analysis, 30 μg of proteins were loaded onto 4–12% polyacrylamide gel followed by electrotransfer to nitrocellulose membrane. Membranes were next incubated with rabbit polyclonal antibodies against Col-1α1 (Cell Signaling, Leiden, Netherlands, Cat. No. 84336), and GAPDH (Cell Signaling, Leiden, Netherlands, Cat. No. 2118), or mouse monoclonal antibodies against fibronectin (Abcam, Cambridge, United Kingdom, Cat. No. ab6328), overnight at 4 °C followed by incubation with IgG-HRP-conjugated anti-rabbit or anti-mouse secondary antibodies (Amersham, GE Healthcare, Illinois, USA, Cat. no. NA934V) for 1 h at room temperature. The immune complexes were visualized by enhanced chemiluminescence (ECL SuperSignal™ West Dura Extended Duration Substrate, Thermo Fisher Scientific, Massachusetts, USA, Cat. No. 34075) with the ChemiDoc™ Touch Imaging System (BIORAD, Hercules, California, USA). The intensity of protein bands was quantitated by ImageJ Software (Schneider, C. A.; Rasband, W. S. & Eliceiri, K. W. (2012), "NIH Image to ImageJ: 25 years of image analysis", Nature methods 9(7): 671-675, PMID 22930834).

### 2.3. Cell Survival with Atropine

Initially, 5 × 10^3^ HCE-F/well were seeded in 96-well plates and left to adhere, overnight, in complete culture medium. In a first set of experiments the following treatments were carried out in SFM: atropine (1, 1.7, and 3.4 mM), for 24, 48, and 72 h. In a second set of experiments, HCE-F cells were treated with 0.15% HA (HMW: 1.8 MDa; Fidia pharmaceutical SpA, Abano Terme, Italy), 0.5% colostrum (NZpurehealth, Auckland, New Zeland), 0.5% 2-Fucosyl-Lactose (Carbosynth, Berkshire, UK, cat. No. OF06739) and two mixes (Mix A: 0.15% HA + 0.5% colostrum; Mix B: 0.15% HA + 0.5% 2FL) in the presence of atropine 1 mM; the medium with the supplements was changed every day for 4 days. Cytotoxicity was measured by the MTT method with the cell counting kit-8 assay (CKK8, Sigma-Aldrich, St. Louis, Missouri, USA, Cat. No. 96992) according to the manufacturer’s protocol.

## 3. Results

### 3.1. ECM Production in Scleral Fibroblasts

**Figure 1:**
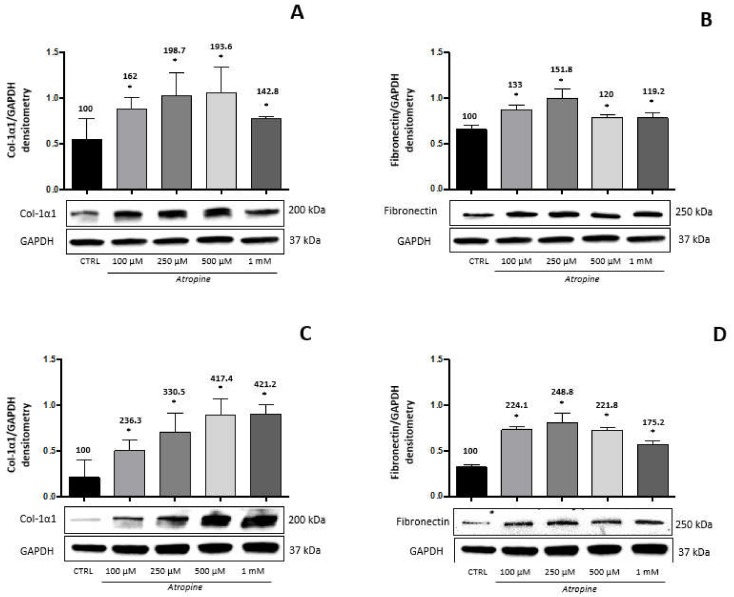
Atropine effects on collagen and fibronectin production by HSF cells. Western immunoblot analysis of collagen type-1α1 (Col-1α1) and fibronectin in HSF lysates treated with atropine at different doses as indicated for 24 and 48 h. A representative Western immunoblot is shown at the bottom of each panel, and the quantitative analysis is reported in the histogram above. Numbers on bars indicate the percentage value with respect to the control, set at 100%. Each bar represents the average value ± SD of three different experiments: (**A**) Col-1α1 24 h; (**B**) Fibronectin 24 h; (**C**) Col-1α1 48 h; and (**D**) fibronectin 48 h. *p < 0.05 vs. CTRL. One-way ANOVA, followed by Tukey’s test.

Starting from the hypothesis that the elongation of the eye globe in myopia should involve the biosynthesis of ECM proteins in the sclera, and that atropine might interfere with this process, we treated HSF cells with increasing doses of atropine (from 0.1 to 1.0 mM) for 24 and 48 h, and measured the amount of collagen and fibronectin in cell lysates. Figure 1 shows that both collagen type 1-α-1 (Figure 1A,C) and fibronectin (Figure 1B,D) biosynthesis were increased at 24 h (Figure 1A,B) and 48 h (Figure 1C,D) of treatment with atropine, in a dose-dependent fashion more evident for collagen, while for fibronectin a plateau seemed to be reached already at 0.1 mM. The maximum increase, respectively, at 24 and 48 h for collagen at the dose of 500 μM was 193% and 417%, while for fibronectin at the dose of 250 μM was 151.8% and 248.8%.

**Figure 2:**
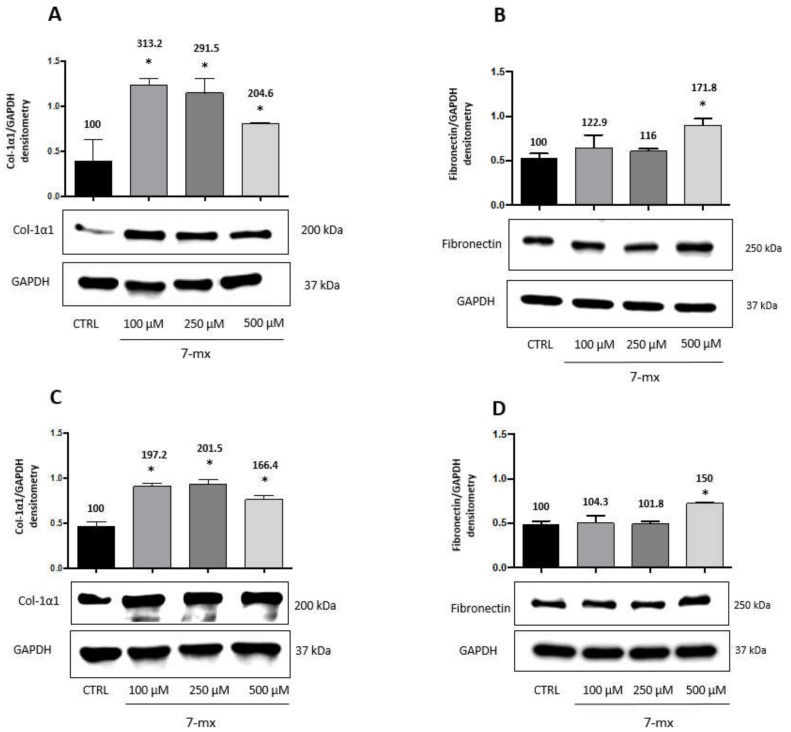
7MX effects on collagen and fibronectin production by HSF cells. Western immunoblot analysis of collagen type-1α1 (Col-1α1) and fibronectin in HSF lysates treated with 7MX at different doses as indicated for 24 and 48 h. A representative Western immunoblot is shown at the bottom of each panel, and the quantitative analysis is reported in the histogram above. Numbers on bars indicate the percentage value with respect to the control, set at 100%. Each bar represents the average value ± SD of three different experiments: (**A**) Col-1α1 24 h; (**B**) Fibronectin 24 h; (**C**) Col-1α1 48 h; and (**D**) fibronectin 48 h. *p < 0.05 vs. CTRL. One-way ANOVA, followed by Tukey’s test.

In agreement with these results, we show in Figure 2 that the adenosine receptor antagonist 7MX, also known to retard myopia development [14], had similar effects on HSF, increasing both collagen type 1-α-1 (Figure 2A,B) and fibronectin (Figure 2C,D) at 24 h (Figure 2A,C) and 48 h (Figure 2B,D), although fibronectin elevation was evident only at the highest dose of 0.5 mM. The maximum increase, respectively, at 24 and 48 h at doses of 100 and 250 μM for collagen was 313% and 201%, while for fibronectin at the highest dose of 500 μM was 171.8% and 150%. A plateau in collagen production was reached already at the lower dose of 0.1 mM, suggesting an effect maybe stronger than that of atropine.

### 3.2. ECM Production in Choroidal Fibroblasts

**Figure 3:**
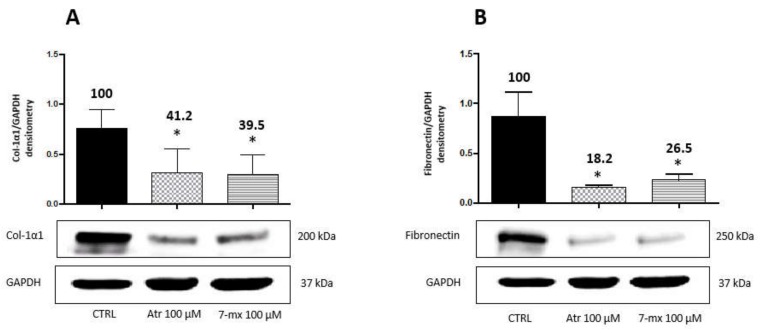
Atropine and 7MX effects on collagen and fibronectin production by HCOF cells. Western immunoblot analysis of collagen type-1α1 (Col-1α1) and fibronectin in HCOF lysates treated with atropine or 7MX at 100 μM for 48 h. A representative Western immunoblot is shown at the bottom of each panel, and the quantitative analysis is reported in the histogram above. Numbers on bars indicate the percentage value with respect to the control, set at 100%. Each bar represents the average value ± SD of three different experiments: (**A**) Col-1α1 48 h; and (**B**) fibronectin 48 h. *p < 0.05 vs. CTRL. One-way ANOVA, followed by Tukey’s test.

Since the choroid is a tissue adjacent to the sclera and the retina, which also appears to be involved in myopia development [8], we also analyzed the response of human choroidal fibroblasts (HCOF) to atropine or 7MX. Based on previous results with HSF, HCOF were treated with either drug at 0.1 mM for 48 h. Figure 3 shows that, opposite to what happens in HSF, both atropine and 7MX produced in this cell line a strong decrease in the biosynthesis of collagen (around 60%) and fibronectin (around 80%), suggesting that atropine and 7MX can produce their effects on myopia through different mechanisms depending on the district in which they operate.

### 3.3. Atropine Toxicity Is Blunted by Colostrum and Fucosyl-Lactose

**Figure 4:**
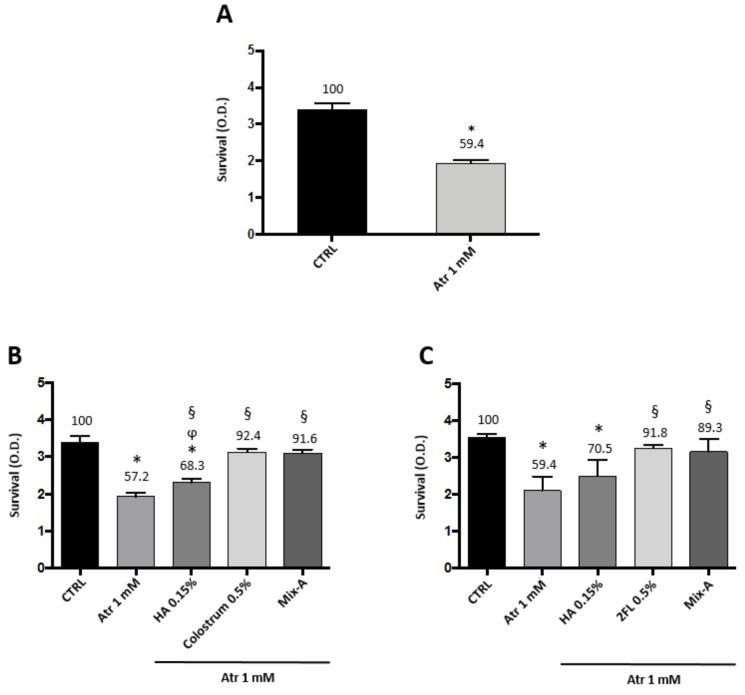
Atropine toxicity on corneal epithelial cells is blunted by colostrum and 2-fucosyl-lactose. HCE-F corneal epithelial cells viability assay in presence of atropine and the protective effects of colostrum or 2-fucosyl-lactose: (**A**) Cell survival after four-day treatment with atropine 1 mM; (**B**) cell survival after four-day treatment with atropine 1 mM in presence of 0.15% HA, 0.5% colostrum, and a combination of 0.15% HA and 0.5% colostrum (Mix-A); and (**C**) cell survival after four-day treatment with atropine 1 mM in presence of 0.15% HA, 0.5% 2-fucosyl-lactose, and a combination of 0.15% HA and 0.5% 2-fucosyl-lactose (Mix-B). Values represent average ± SD of triplicate wells. * *p* < 0.05 vs. CTRL, § *p* < 0.05 vs. atropine 1 mM, ϕ *p* < 0.05 vs. colostrum 0.5%. One-way ANOVA, followed by Tukey’s test.

Low dose atropine given topically as eye drops is by far the most used drug treatment to try and slow down myopia progression in children. The treatment has to be given daily, for periods of time usually higher than one year [15]. Atropine is known to interfere with lacrimation [16,17], and therefore might pose treated patients at risk of dry eye [12]. Therefore, we examined the effects on primary human corneal epithelial cells (HCE-F) of a relatively long-term treatment (96 h) with low dose atropine (1 mM, corresponding to a 0.03% solution). Data illustrated in Figure 4A show indeed that after 96 h with the continuous presence of atropine there was a 40% loss of vitality in HCE-F cells. However, the simultaneous presence in the culture medium of either 0.5% of bovine colostrum (Figure 4B) or 0.5% 2FL (Figure 4C) prevented such toxicity, keeping the survival at values around 90% of control levels. HA alone was only partially effective (70% of control levels), improving survival with respect to atropine (60% of control levels), but still lower than untreated control. The mix with 0.15% HA of colostrum at 0.5% or 2FL at 0.5% was equally effective (90% of control levels) as the single components alone. This toxicity of atropine, which is clearly manifest on corneal epithelial cells at the therapeutic dose of 1 mM, was not apparent on scleral or choroidal fibroblasts until 48 h (not shown), but it might explain why the dose effect on collagen and fibronectin synthesis was not further increased at doses higher than 0.5 mM.

## 4. Discussion

It is nowadays evident that myopia is strongly correlated with the axial length of the eye and not with its refractive power [18]. The growth of the eye globe is a complex event, which appears to be regulated by signals coming from the retina and the choroid, independent from the brain [19,20]. Some described chemical mediators of this signaling mechanisms are dopamine [21], TGFβ [22], and melatonin [23]. Inflammatory cytokines have also been detected, and it has been speculated that they might be involved in the development and progression of high myopia and myopic retinopathy [24].

However, whatever the triggering signals, growth of the eye globe leading to axial elongation must involve the scleral tissue and its stroma, which lead the growth of the eye globe. Collagen makes up roughly 90% of the scleral proteins, with type I collagen showing the highest expression (>99%) of the many collagen subtypes [25]. All the evidence thus far indicates that a decreased amount of collagen type I—due in part to a reduced biosynthesis and in part to increased degradation—is responsible of the thinning of the sclera and its abnormal and progressive elongation [26]. Therefore, it is conceivable that a main way to arrest myopia progression is to strengthen the scleral stroma, by increasing its collagen content. Indeed, what we found and showed in this study is that the effect of atropine on human scleral fibroblasts is to dramatically enhance their type I collagen production, which is in line with the expected efficacy of atropine to slow down myopia progression. Similar results were described in an in vivo model system of guinea pig myopia induced by form deprivation [27]. In this model, the posterior scleral thinning was accompanied by a significant decrease in posterior scleral collagen type I mRNA; atropine treatment attenuated the scleral remodeling associated with myopia and significantly increased collagen type I mRNA expression. Consistently with the above results, we show here for the first time that also 7MX—which is known to work against myopia [10,11]—has the effect of enhancing type I collagen biosynthesis in HSF cells, although to a slightly lesser extent than atropine.

Fibronectin is a prominent glycoprotein of basement membranes (BM), interacting with specific cell surface integrin receptors and triggering growth and survival signals [28]. It is present in all layers of Bruch’s membrane [29], which is the BM produced by RPE cells, and is also involved in myopia progression [30]. Therefore, our findings here reported for the first time that both atropine and 7MX can also stimulate fibronectin production by HSF cells might have implications that go beyond the structure of the sclera (likely, fibronectin does not contribute much to its rigidity or elasticity). In fact, if a similar effect on the production of ECM proteins by atropine or 7MX can be confirmed also on RPE cells, this might further suggest that the structure of the Bruch’s membrane indeed has a role in myopia development and progression.

Unexpectedly, atropine and 7MX exerted an opposite effect on HCOF cells, since a decrease of both type I collagen and fibronectin expression was observed. The response of HOCF cells to the two antagonist mediators suggests that those cells express the cognate receptors. Adenosine receptors responsive to 7MX were already reported in the choroid of rhesus monkeys [31], and muscarinic receptors were detected in the choroid of tree shrews [32] and chickens [33]. None of these receptors varied expression in correlation with myopia development. However, in tree shrews, a network of choroidal gene expression changes has been supposed to generate signals impinging on scleral fibroblast gene expression regulating the axial elongation rate, thus suggesting an active role for the choroid in the signaling cascade from retina to sclera [34]. Decreased choroidal thickness and choroidal blood perfusion were correlated with progressing myopia in a guinea pig model [35] and humans [36]. It is therefore tempting to speculate that, since scleral hypoxia has been proposed as a target for myopia control [8], the decreased production of ECM proteins triggered by atropine and 7MX in choroid fibroblast could favor blood perfusion through a less dense ECM, thus rescuing the scleral tissue from hypoxia.

Atropine effects are mediated though its antagonist action on muscarinic receptors. Therefore, a first hypothesis is that the opposite effects of atropine on HSF and HOCF cells might be due to a differential distribution and localization of muscarinic receptor subtypes in ocular tissues. It has been reported that the suppression of M2 receptors increases collagen I production in the sclera of KO mice, making them resistant to myopia induction [37]. In tree shrews, the expression of M2 receptors appears to be different in the sclera and the choroid [32]. In humans, corneal and conjunctival cells express different patterns of muscarinic receptors [38]. Moreover, being the muscarinic receptors coupled to G proteins, their signal transduction pathway may also vary in different cell types, depending on their expression levels and physiological status of the cells [27,39,40]. A similar situation might exist also for 7MX receptors in ocular tissues [31].

Low dose atropine and 7MX are widely recognized as efficient treatments to retard myopia progression [41]. Both drugs exert their effects after several months and even years of treatment. A rebound effect has been described upon interruption of atropine treatment, even though the final result in terms of axial elongation is still in favor of treatment [42]. While 7MX is given orally, atropine is administered topically as eye drops. Due to the long-term treatment schedule, toxicity can be an issue. Both low dose atropine and 7MX treatment have been reported to be safe [14,43]. However, a concern over possible dry eye development after long-term atropine treatment has been raised [12]. It is known that repeated instillations (three times over 12 h) of 1.0% atropine sulfate is enough to induce dry eye in the rabbit [44], because of an inhibitory effect on stimulated tear secretion from the main lacrimal gland [16]. Moreover, cytotoxic effects in vitro of 0.03% atropine on human corneal epithelial cells have been reported [45]. Accordingly, our data also indicate that primary human corneal epithelial cells (HCE-F) grown four days in the continuous presence of atropine show signs of toxicity and decreased survival.

Severe cases of dry eye or corneal ulcerations can be treated with biological derivatives rich of growth and survival factors, such as autologous serum eye drops [46] or cord blood serum eye drops [47]. Another biological product with similar characteristics, but easier to obtain, is bovine colostrum. This is generally obtained during the first few hours after cow’s delivery, so that it contains significant and reproducible amounts of bioactive factors, including cytokines, immunomodulating factors, growth factors, and immunoglobulins [48]. The 2FL is the most abundant oligosaccharide in colostrum and milk of lactating mothers [49]. Both colostrum [50] and 2FL [16] have been used as eye drops to treat ocular surface diseases. We showed in this study that the addition of either bovine colostrum or 2FL can efficiently blunt the toxic effects of long-term (four days) atropine treatment of HCE-F cells, significantly better than 0.15% hyaluronic acid (normally present in artificial tears to treat dry eye conditions).

## 5. Conclusions

The data presented in this paper corroborate the hypothesis that one of the atropine effects on slowing down myopia depends on its ability to stimulate ECM (collagen and fibronectin) biosynthesis in scleral fibroblast cells, thus thickening the scleral tissue and reducing its elasticity and tendency to prolapse. Moreover, we show that atropine can work differently on cells of different origin, since it decreased ECM biosynthesis in choroidal fibroblasts. This finding might hint to another effect of atropine on slowing down myopia progression, i.e., an improvement of scleral blood perfusion through the choroid, due to a higher permeability of its ECM. Finally, the finding that atropine toxicity could be prevented by the simultaneous presence of colostrum or its main oligosaccharide component (2FL) should suggest that eye drops with either one of these constituents could be used in myopic children subjected to chronic treatment with atropine.
